# A Case of Gaseous Colic

**Published:** 1857-01

**Authors:** Thos. S. Denny


### ARTICLE II.

*A. Case of Gaseous Colic.* By Thos. S. Denny, M. D.

In one of the recent numbers of your Journal I observed a case of "Gaseous Colic," reported by Dr. W. T. Grant of Wrightsboro'.

I recollect a case somewhat similar, though more violent, which occurred in my practice sometime since.

The patient fell as suddenly as if from an attack of apoplexy, which resulted, as he afterwards informed me, from the extreme intensity of the pain.

I saw him immediately after the attack; he was nearly insensible, and it was with difficulty that the pulse could be perceived at the wrist. I immediately administered a strong dose of brandy, laudanum and ess. menth. pip. The effect was almost instantaneous—an immense quantity of wind was discharged from the stomach, which afforded, of course, instant relief. The pain, however, recurred in about half an hour, and required one or two more doses of the same kind, together with the warm bath, (coxluævium,) to complete the cure.

This case was accompanied, on the recurrence of the pain, and I presume on the first attack, likewise, by a violent spasmodic erection of the penis, adding much to the suffering of the patient. Over this latter symptom the warm bath seemed to exercise almost magical effect; the patient slept immediately after its use, and was as well as usual the next morning, with the exception of some debility, natural after such a violent excitement of the system.

As to the *cause* in this instance, I am now at a loss, if I was acquainted with it at the time.

